# The Entry and Egress of Monocytes in Atherosclerosis: A Biochemical and Biomechanical Driven Process

**DOI:** 10.1155/2021/6642927

**Published:** 2021-07-08

**Authors:** Hongyan Kang, Xinyu Li, Kewen Xiong, Zhiyun Song, Jiaxin Tian, Yuqiao Wen, Anqiang Sun, Xiaoyan Deng

**Affiliations:** ^1^Beijing Advanced Innovation Center for Biomedical Engineering, Key Laboratory for Biomechanics and Mechanobiology of Ministry of Education, School of Biological Science and Medical Engineering, Beihang University, Beijing, China 100083; ^2^Artificial Intelligence Key Laboratory of Sichuan Province, School of Automation and Information Engineering, Sichuan University of Science and Engineering, Zigong, Sichuan, China 643000

## Abstract

In accordance with “the response to injury” theory, the entry of monocytes into the intima guided by inflammation signals, taking up cholesterol and transforming into foam cells, and egress from plaques determines the progression of atherosclerosis. Multiple cytokines and receptors have been reported to be involved in monocyte recruitment such as CCL2/CCR2, CCL5/CCR5, and CX3CL1/CX3CR1, and the egress of macrophages from the plaque like CCR7/CCL19/CCL21. Interestingly, some neural guidance molecules such as Netrin-1 and Semaphorin 3E have been demonstrated to show an inhibitory effect on monocyte migration. During the processes of monocytes recruitment and migration, factors affecting the biomechanical properties (e.g., the membrane fluidity, the deformability, and stiffness) of the monocytes, like cholesterol, amyloid-*β* peptide (A*β*), and lipopolysaccharides (LPS), as well as the biomechanical environment that the monocytes are exposed, like the extracellular matrix stiffness, mechanical stretch, blood flow, and hypertension, were discussed in the latter section. Till now, several small interfering RNAs (siRNAs), monoclonal antibodies, and antagonists for CCR2 have been designed and shown promising efficiency on atherosclerosis therapy. Seeking more possible biochemical factors that are chemotactic or can affect the biomechanical properties of monocytes, and uncovering the underlying mechanism, will be helpful in future studies.

## 1. Introduction and Overview

Cardiovascular diseases (CVDs) are the leading cause of morbidity and mortality worldwide [1]. Data from WHO shows (2018) ischemic heart disease and stroke, as the world's biggest killers in the last 15 years, accounted for a combined 15.2 million deaths in 2016. As the main cause of most cardiovascular diseases [2], atherosclerosis can lead to many clinical manifestations including myocardial infarction, cerebrovascular accident, and peripheral vascular disease. Although the etiology of atherosclerosis is complex and closely associated with personal genetic, dietary, lifestyle, metabolic, and immune function, it is proved that macrophages play an important role in the development of atherosclerotic lesions, as their entry into the subendothelial space guided by inflammation signals, taking up cholesterol and transforming into foam cells, as well as exit from tissues determine the progression of atherosclerosis [3, 4]. However, the mechanism that regulates the entry and egress of macrophages from plaques remains largely unknown [5]. In the present review, we collated cytokines, receptors, and biomechanical factors that affect the entry and egress of monocytes into and from the atherosclerotic plaque, and we also discussed potential therapeutic approaches targeting the migration of monocytes in the latter section.

## 2. Atherosclerosis

### 2.1. The Initiation and Progression of Atherosclerosis

Atherosclerosis is a disease of chronic inflammation that is associated with multiple cells including monocytes, endothelial cells (ECs), vascular smooth muscle cells (VSMC), monocytes derived macrophages, and dendritic cells and regulatory T cells (TREG), and could be distinguished by the presence of cholesterol-engorged macrophages in arterial plaques [6]. Initially, when the endothelium is perturbed by risk factors like disturbed blood flow, high plasma low-density lipoprotein (LDL) concentration, hypertension, toxins from cigarette smoke [7], or bacterial antigens and membrane components, the endothelial cells can be activated and release a range of chemokines, like intercellular adhesion molecule-1 (ICAM-1) and vascular cell adhesion molecule-1 (VCAM-1). As a result, monocytes are recruited to the arterial wall; then, they differentiate into macrophages, which take up LDL, and eventually become foam cells in the intima [8]. Foam cells can contribute to plaque instability by secreting inflammatory molecules and extracellular matrix-degrading proteases [9]. In the lipid core, apoptotic foam cells will go through a process termed efferocytosis that is performed by the phagocytic macrophages mostly to prevent the accumulation of dead cell debris in the lipid core. Unfortunately, in most cases, phagocytic macrophages cannot gulf all dead cells for a long term, so a necrotic core formed. As the necrotic core grows up and the fibrous cap becomes thinning, the vulnerable plaque will be prone to rupture, subsequently leading to thrombosis formation in the coronary arteries [4].

### 2.2. Cytokines Involved in the Initiation and Progression of Atherosclerosis

In the processes of atherosclerosis initiation and progression, cytokines have a profound influence at all stages by regulating related cell migration [10]. Until now, eighteen signaling chemokine receptors have been identified, and over 50 chemokine ligands are found in humans [11]. The cytokines involved in atherosclerotic plaque formation include interleukin- (IL-) 1, IL-2, IL-3, IL-4, IL-5, IL-6, IL-9, IL-10, IL-12, IL-13, IL-15, IL-17, IL-18, IL-20, IL-25, IL-27, IL-33, IL-37, tumor necrosis factor- (TNF-) *α*, TGF-*β*, interferon- (IFN-) *α*, IFN-*β*, IFN-*γ*, growth differentiation factor- (GDF-) 15, granulocyte colony-stimulating factor (G-CSF), granulocyte-macrophage colony-stimulating factor (GM-CSF), macrophage-colony stimulating factor (M-CSF), and tumor necrosis factor-related apoptosis-inducing ligand (TRAIL) [10]. Whereas chemokines (family of small cytokines) involved in atherosclerosis advancements are CC-chemokine ligand (CCL) 2, CCL3, CX3C-chemokine ligand (CXCL) 4, CCL5, CXCL1, CX3CL1, CCL17, CXCL8, CXCL10, CCL20, CCL19, CCL21, and macrophage migration-inhibitory factor [10]. In the present review, we will give a detailed description of the chemokines or cytokines that are related to monocytes' entry and egress into and from plaques in the progression of atherosclerosis.

## 3. Cytokines/Receptors and Other Biochemical Factors Involved in Monocyte Migration

It has been realized that interventions to encourage the egress of monocytes from plaques may be a promising therapeutic approach for atherosclerosis treatment [4]. However, a better understanding of cytokines that regulate monocyte migration and the underlying mechanism is the first challenging step.

### 3.1. Monocyte Subsets

**Figure 1:**
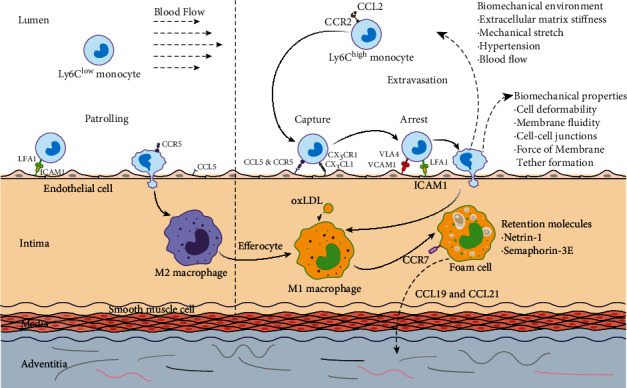
Cytokines, receptors, and biomechanical factors affecting the entry and egress of monocytes in the progression of atherosclerosis. The accumulation of Ly6C^high^ monocytes in the nascent atherosclerotic plaque is triggered by the molecules secreted by endothelial cells, such as CCL2, CCL5, and CXC3L1. The interaction between lymphocyte function-associated antigen 1 (LFA1) or very late antigen 4 (VLA4) and endothelial adhesion molecules, such as intercellular adhesion molecule 1 (ICAM1) and vascular adhesion molecule 1 (VCAM1), facilitates the firm adhesion formation. After entering the intima, Ly6C^high^ monocytes differentiate into M1-type macrophages that take up LDL and finally become foam cells. On the other hand, macrophages can be released from the plaque by the interaction of CCR7, CCL19, and CCL21. However, the egress process may be inhibited by some retention factors secreted by foam cells like Netrin-1 and semaphorin 3E. The whole process of monocytes entry and egress could be regulated by their biomechanical properties such as the membrane fluidity, the deformability, cell-cell junctions, and force of the membrane tether formation, as well as the surrounding biomechanical environment including the stiffness of the extracellular matrix, stretch from the neighboring vein, blood flow-induced shear stress, and hypertension. As for Ly6C^low^ monocytes, CCL5 plays a major role in their recruitments into the intima. Thereafter, the Ly6C^low^ monocytes differentiate into M2-type macrophages, which can phagocytose the apoptotic M1 macrophages and stabilize the plaque.

In mice, monocytes in the blood are generally classified into two types, Ly6C^high^ and Ly6C^low^ (Figure 1). Ly6C^high^ monocytes express high levels of CC chemokine receptor- (CCR-) 2 and are believed to be proinflammatory for their recruitment to sites of inflammation, which are accounted for 50% of the whole monocytes pool, and their levels increase in hyperlipidemia [12]. These monocytes can infiltrate into tissues, differentiate into Tip-DC, M1-type, or classically activated macrophages, mediate inflammation and proteolysis by engulfing pathogens, and produce antibacterial products [13]. It should be noted that the Ly6C^high^ monocytes derived M1 macrophages are enriched in progressing plaques [8], and their activation of the NADPH oxidase system for the removal of pathogens during infection can induce tissue damage and impair wound healing. Corresponding to human monocytes, Ly6C^high^ monocytes in mice are thought to resemble CD14^+^ CD16^–^ monocyte subtypes in humans. However, in humans, the CD14^+^ CD16^–^ monocytes account for 95% of the whole monocytes pool, which is much higher than the proportion of Ly6C^high^ monocytes in mice.

On the contrary, Ly6C^low^ monocytes with a typical high expression of CX3C-chemokine receptor 1 (CX3CR1) while no expression of CCR2 are believed to carry out a homeostatic function as they patrol the luminal side of the endothelium of small blood vessels. Moreover, Ly6C^low^ monocytes correspond to the CD14^low^ CD16^+^ monocyte subset in humans, which are believed to be the precursor cells of M2 macrophages. The M2 macrophages are thought to be anti-inflammatory, because they have an anti-inflammatory cytokine profile, and they can phagocyte apoptotic M1 macrophages [8], contributing to the resolution of inflammation; additionally, they have an increased secretion of collagen, which promotes tissue repair [14].

### 3.2. Cytokines/Receptors Responsible for Monocyte Recruitment

#### 3.2.1. CCL2/CCR2

The first molecule that has been investigated in atherosclerosis pathogenesis is chemokine ligand 2 (CCL2), also known as monocyte chemoattractant protein-1 (MCP-1), and it works with receptor chemokine receptor type 2 (CCR2). CCL2 is a chemokine produced by a variety of cell types, including monocytes, macrophages, smooth muscle cells, and endothelial cells within atherosclerotic plaques [15]. At the molecular level, CCL2 is a member of C-C chemokines which commonly contains four completely conserved cysteine residues with two disulfide bonds within these small proteins. At the amino-terminal region, residues 1–6 are essential for chemoattractant activity, among which the Asp-3 has been proved to play a key role in particular [16]. Whereas the other N-terminal side is less exposed by binding with glycosaminoglycans (GAGs), being “buried” in the dimer and even tetramer form [17], resulting in an inhibitory effect on the binding of the receptor [18], which contributes to CCL2 receptor binding specificity and its activity regulation.

CCL2 is encoded by the small inducible CCL2 gene, which is located on chromosome 17q11 [19]. The expression of CCL2 can be elevated at the transcription level by multiple stimuli, including tumor necrosis factor-alpha (TNF-*α*), interferon-gamma (IFN-*γ*), platelet-derived growth factor (PDGF), and stress factor, while inhibited by retinoic acid, glucocorticoids, and estrogen [20]. These different stimuli have one thing in common that they can affect the proinflammatory nuclear factor kappa B (NF-*κ*B), a key transcription factor [21], which in turn regulates the gene expression of CCL2 [19].

A number of animal studies have demonstrated that high levels of CCL2 are associated with the elevated risk of atherosclerosis, which is consistent with the observation in clinical patients with acute coronary syndromes in which patients with the higher level of CCL2 tested [22] have the higher risk of getting bad prognosis [23]. In according to this, atherosclerosis models like CCL2 gene knock out in LDL receptor–deficient mice [24] or CCR2 deprivation models generated by crossing mice that lack CCR2 with apo E-null mice which develop severe atherosclerosis [25] showed a promising effect on atherosclerosis prevention. In fact, this phenomenon has been reported in mice that the blockade of CCL2 by transfecting an N-terminal deletion mutant to the CCL2 gene limited the progression of preexisting atherosclerotic lesions in the aortic root of hypercholesterolemic mice [26].

In the initiation and progression of atherosclerosis, CCL2 functions as a direction cues trafficking of monocytes across the endothelium [27]. After binding with its receptor CCR2 expressed on Ly6C^high^ monocytes, it guides these cells to migrate into the subendothelial space, which is believed to be one of the earliest steps in atherogenesis [28]. More specifically, CCL2 exerts its effects through binding to G-protein coupled receptors CCR2 on the Ly6C^high^ monocytes or other target cells. Activated receptor CCR2 then can trigger a series of cellular activities, i.e., the monocytes inositol triphosphate formation, intracellular calcium release, and protein kinase C activation [27], which lead to changes in the cytoskeleton and in adhesive interactions between the extracellular matrix and cell surfaces to produce locomotion [18]. Moreover, studies have substantiated that CCR2 can also promote the bone marrow release of Ly6C^high^ monocytes to the blood circulation, thus, enhancing the recruitment of Ly6C^high^ monocytes [29].

#### 3.2.2. CCL5/CCR5 and CX3CL1/CX3CR1

Except for CCL2/CCR2, CCL5/CCR5 and CX3CL1/CX3CR1 are also involved in the recruitment of monocytes [13]. It has been shown that combined inhibition of these three chemokines led to a 90% reduction in atherosclerosis plaque in Apo E-deficient mice [30]. Furthermore, in the same experiment, the reduction size of the plaque in the CX3CR1-deficient, CCL2-deficient, and CCL2/CX3CR1-deficient mice was 28%, 36%, and 48%, respectively, with respect to the ordinary Apo E-deficient atherosclerosis model, which suggested that CCL2/CCR2 and CX3CL1/CX3CR1 are independent modulators in atherosclerosis progression. In fact, CCL2/CCR2 mainly functioned in Ly6C^high^ monocyte trafficking [31], extravasation from the bone marrow, and favoring rolling and infiltration of circulating monocytes through the endothelium layer. However, all these processes are integrin-activation dependent. On the contrary, CX3CL1 and its specific receptor CX3CR1 allow firm adhesion of monocytes on endothelial and smooth muscle cells independently of integrin activation [32]. Moreover, some researchers believe that CX3CR1 promotes Ly6C^high^ cell survival from the bone marrow by inhibiting their apoptosis, thus, influence the number of monocytes entering into the plaques [14].

On the other hand, the entry of Ly6C^low^ monocytes to plaques is CX3CR1 independent [31]. In this process, CCR5 was shown to be critical, and inhibition of CCR5 signaling led to a marked reduction in the number of circulating monocytes especially at the later stage of atherosclerosis, which correlated with the reduced lesion size [33]. The mechanism may be that CCR5/CCL5 plays a major role in Ly6C^low^ recruitments into the intima. After that, the Ly6C^low^ monocytes differentiate into M2-type macrophages [14], which can phagocytose the apoptotic M1 macrophages, contributing to the resolution of inflammation and stabilize the plaque [8, 34].

### 3.3. Cytokines/Receptors Involved in the Egress of Macrophages from the Plaque

The monocyte recruitment and its entry into the subendothelial space is one of the early events in atherosclerosis initiation. After transforming into macrophages, these cells take up LDL and give rise to foam cells, which stay there and drive the progression of atherosclerosis. Therefore, looking for factors affecting the egress of macrophages from the plaque may be a promising strategy for the regression of established atherosclerosis. Fortunately, people discovered that under the regulation of CCL19, CCL21, and their receptor CCR7, macrophages can exit from the atherosclerotic lesions [35].

CCR7 is one of the most prominent chemokine receptors in the adaptive immune system and play important roles in promoting homing of T cells and DCs to lymphoid tissues [11], facilitating the egress of macrophage from the atherosclerotic region [36], and directing the entering and positioning of these cells within secondary lymphoid organs (SLOs). CCR7 can be expressed in many immune cells including subsets of thymocytes, T cells, B cells, DCs, macrophages [37], and neutrophils. The expression of CCR7 in macrophage is essential for decreasing the macrophage amount in plaques and promoting the regression of atherosclerosis, by interacting with its specific ligands CCL19 and CCL21 [38, 39].

CCL19 and CCL21 are chemokines that widely exist in lymphoid organs and are generally considered “homeostatic.” Unlike many other chemokines induced by inflammation, they are produced by stromal cells within primary and secondary lymphoid organs under normal physiological conditions. CCL21 can also be produced by lymphatic endothelial cells (LECs) in peripheral tissues. As the ligand of CCR7, the structures, functions, and regulatory mechanisms of CCL 19 and CCL21 are quite different. CCL21 was thought to be a predominantly matrix and endothelial cell-bound chemokine, as it has an unusual extended C-terminal tail that shows high binding affinity to glycosaminoglycans (GAGs) and other elements in the extracellular matrix. These matrix-bound CCL21 promote both chemotactic migration of cells and cell adhesion [40], particularly under shear forces. By contrast, CCL19 lacks this extended C-terminal tail and is more available locally in a soluble form. It should be noted that CCL21 could also exert effects in a soluble form the same as CCL19, while this soluble form only induces chemotaxis but no adhesion.

Although CCL19 and CCL21 are recognized as homeostatic chemokines in physiological condition, they can actually be induced in certain inflammatory situations. Inappropriate expression of CCL19 and CCL21 will bring dramatic effects, resulting in tissue-specific tertiary lymphoid organ formation in both CCL21a and CCL21b transgenic mice [41]. The production of CCL21 can be induced in both lymphotoxin-dependent and lymphotoxin-independent manner [42], while the induction of CCL19 seems to be lymphotoxin-dependent. In lymphoid stroma, lymphotoxin induces CCL19 and CCL21a predominately, and this induction is associated with the existence of lymphotoxin. It has been demonstrated that both CCL19 and CCL21a are absent in secondary lymphoid organs in mice which lack membrane lymphotoxin or TNF-*α*, due to the impairment of these chemokines mediated interactions between lymphoid tissue inducer cell and stromal organ cells. In addition, as we mentioned before, inflammatory signaling can induce CCL21a production, for example, subcutaneous injection of IL-1b and TNF-*α* increased the mRNA level of CCL21 in lymphatic endothelial cells [43]. However, the expression of CCL21b in peripheral tissues is lymphotoxin-independent. It should be noted that CCX-CKR expressed on stromal cells also has a lower binding affinity for CCL19 and CCL21, while it does not mediate cell migration. By competitive binding to CCL21 and CCL19, CCX-CKR can weaken the role of CCR7.

In order to investigate the role of CCR7/CCL19/CCL21 in atherosclerosis progression, the plaque-containing arterial segments from apo E-deficient mice were transplanted into the wild-type recipient normolipidemic mice to induce an atherosclerosis regression. Results showed that the plaque size decreased by 40%, accompanied by a 75% reduction of the foam cell content in the plaque, with increased mRNA levels of liver X receptor and cholesterol efflux factors ABCA1 and SR-BI in foam cells, reduced expression levels of VCAM or MCP-1, and enhanced mRNA and protein levels of chemokine receptor CCR7 in wild type recipient normolipidemic mice [35]. On the other hand, abrogation of CCR7 using antibodies against ligands CCL19 and CCL21 resulted in the lesion size and foam cell content in Apo E-deficient mice preserved [35], which substantiated the key role of CCR7 in mediating macrophages egress from the plaques. Furthermore, drugs that activate the nuclear receptor liver X receptors (LXR) have been proved successfully in animal models to induce atherosclerotic lesion regression by upregulating the expression of CCR7 in macrophages [44].

### 3.4. Cytokines Exerting Inhibitory Effects on Migration of Monocytes/Macrophages

#### 3.4.1. Netrin-1

A recent study from van Gils et al. [5] indicated that Netrin-1 and its receptor uncoordinated-5 homolog B receptor (UNC5b) would inhibit the egress of macrophage from the plaque. Netrin-1, a kind of neuronal guidance molecule, helps the nervous system to found the correct neural pathway [9]. It is widely expressed in many cells [45] such as endothelial cells [14] and foam cells. As the chemorepulsive receptor for Netrin-1, Unc5b is predominantly distributed in leukocyte subclasses, including monocytes/macrophages and neutrophils.

The structure of Netrin-1 is similar to the extracellular matrix protein laminin. Its amino-terminal contains two domains: domain IV and domain V, which can bind to the Deleted in Colorectal Cancer (DCC) and UNC5 families receptors [46]. Different receptors binding at the N-terminal will result in different effects. For example, binding to DCC cell surface receptors, some neurons are attracted; while with UNC5 receptors, other neurons are excluded. The sequence at the remaining carboxy-terminal of Netrin-1 (the C-domain) is enriched in basic amino acids.

Apart from its function acts as a signal for neuron migration, Netrin-1 can play an important role in atherosclerosis. It has been found that Netrin-1 and one of its receptors UNC5b were expressed robustly in atherosclerotic plaques in vitro and in vivo [5]. Moreover, Netrin-1 secreted by foam cells exerts different effects on the monocytes and coronary artery smooth muscle cells: it inactivates macrophage migration and prevents its egress from the plaque simultaneously, while enhances the chemoattraction of coronary artery smooth muscle cells, thus, induces SMCs recruitment into the intima and promotes lesion progression. It has been proved in mice that deletion of Netrin-1 in myeloid cells will reduce the size and complexity of atherosclerosis lesion, and this phenomenon is associated with the emigration of macrophages from plaques. Thus, Netrin-1 was established to be an inhibitor of macrophage migration via its receptor UNC5b [9, 47]. The possible mechanism why Netrin-1 and its receptors UNC5b are expressed in atherosclerotic plaque is that atherosclerosis-induced local inflammation causes hypoxia, which in turn mediates transcription factor-1 release, and subsequently, this transcription factor-1 induces the production of Netrin-1 and its receptors UNC5b [48].

By creating a diffusible Netrin-1 gradient across endothelial cell layers [49] (similar to that created by endothelial cell-secreted CCL2), or through the presentation of Netrin-1 binding on the surface of endothelial cells, researchers demonstrated that Netrin-1 could inhibit monocyte chemotaxis. This phenomenon may be attributed to the binding of Netrin-1 to *α*6*β*4 and *α*3*β*1 integrin, as shown on pancreatic epithelial cells [50], thus, inhibit CCL2/CCR2-mediated monocytes trafficking. Nevertheless, further studies are needed to uncover the underlying mechanism.

It should be noted that results from van Gils's work demonstrated Netrin-1 plays a harmful role in the process of atherosclerosis, by inhibiting macrophage egress, thus, retention in the plaque, accelerating the progression of atherosclerosis [5]. However, there are also studies showing Netrin-1 can make a beneficial contribution to the progression of atherosclerosis by preventing monocyte migration into the intima [51, 52]. This discrepancy may be attributed to the stage of atherosclerosis Netrin-1 exerting effects. Another explanation is that the cell sources that produce Netrin-1 [53] are different. In brief, Netrin-1 produced by endothelial cells will inhibit the entry of monocytes into the plaque. While Netrin-1 from macrophages will keep themselves retained in the plaque.

#### 3.4.2. Semaphorin 3A and Semaphorin 3E

There are four major types of neural guidance molecules that control the movement of neurons, the formation of new blood vessels, and the migration of cells outside the nervous system, namely, Netrins, Slits, Semaphorins [54], and Ephrins [55]. Just like Netrin-1, some of them are also involved in the regulation of the immune system [56].

Scientists tried to find out whether there are other neuronal guidance molecules with similar or opposite effects on atherosclerosis [57]. They compared the expression of neuronal guidance molecules in atheroprone (the inner curvature) and athero-protected (the outer curvature) regions of the aortic arch by using custom mRNA arrays. It turns out that the expression of Netrin-1, which is believed to can limit the migration of monocytes, was reduced by 48% in the inner curvature as compared to the outer curvature. Likely, Semaphorin 3A was also downregulated by about 53% in the atheroprone inner curvature with respect to the outer curvature [57]. Protein test found that Semaphorin 3A was expressed on endothelial cells in the athero-protected outer curvature, while there was little to no Semaphoring 3A expression on endothelial cells of the inner aortic curvature. These results suggest that Semaphorin 3A may exert effects similar to Netrin-1 at the early stage of atherosclerosis.

In fact, Semaphorin3A is a secreted protein and one member of a large family of Semaphorins which are commonly composed of 400 to 1000 amino acid residues [58], with a characteristic “sema-domain” comprised of about 500 cysteine-rich amino acids [59]. In a Boyden chamber assay, Semaphorin3A showed inhibitory effect on the migration of freshly isolated human peripheral blood mononuclear cells to CCL1 and CX3CL1 in a dose-dependent manner, which is dependent on its receptor Neuropilin-1 [60]. According to these results, we can speculate that Semaphorin 3 and Netrin-1 may act as a barrier to prevent monocyte adhesion and migration into the arterial intima during the early stage of atherogenesis. However, whether Semaphoring 3A also inhibits the migration of macrophages at the later stage is still unknown.

**Figure 2:**
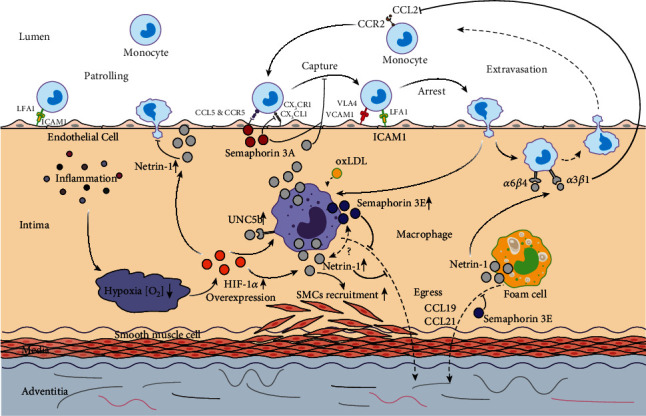
Impact of Netrin-1 and Semaphorins on the entry and egress of monocytes and macrophages in the progression of atherosclerosis. Atherosclerosis-induced local inflammation causes hypoxia, which in turn mediates the release of HIF-1*α*. HIF-1*α* could subsequently upregulate the expression of Netrin-1 and its receptor UNC5b in both macrophages and endothelial cells. Netrin-1 secreted by foam cells and macrophages could inactivate macrophage migration through its receptor UNC5b and prevent its egress from the plaque simultaneously. It could also enhance the recruitment of SMCs and promote lesion progression. However, Netrin-1 secreted by endothelial cell could make a beneficial contribution to the progression of atherosclerosis by preventing monocyte chemotaxis through binding with *α*_6_*β*_4_ and *α*_3_*β*_1_ integrin. Semaphorin 3A inhibits the migration of monocyte to CX3CL1 and its adhesion to endothelial cells. Semaphorin 3E is secreted by macrophages, which could depress the migration of macrophages to chemokines like CCL19 and CCL21.

Another member of the class 3 Semaphorins, Semaphorin 3E, a highly conserved, secreted, and matrix-associated proteins, is highly expressed in macrophages, especially in M1 macrophages, and seems to have a similar function like Netrin-1, contributing to macrophage accumulation in the plaques [61]. It was found that Semaphorin 3E effectively inhibited peritoneal macrophage migration to chemokines like CCL19 and CCL21 in vivo, which plays an important part in promoting inflammatory macrophages to the lymph nodes. An aortic arch transplantation experiment was performed in ApoE−/− mice, in which the aortic arches from high-fat diet-fed ApoE−/− mice were transplanted into either ApoE−/− (progression environment) or WT mice (regression environment); three days after transplantation, mice were sacrificed, and transplanted aortic arches were harvested for microarray test. Results showed the gene expression level of Semaphorin 3E in the plaque in the progression is 6-fold higher than that in the plaque in regression [62], with decreased plaque size and macrophage content. The mechanisms of Netrin-1, Semaphorin 3A, and Semaphorin 3E are shown in Figure 2.

The mechanisms between different cytokines and neural guidance molecules involved in the progression of atherosclerosis were summarized in Table 1.

### 3.5. Other Biochemical Factors Involved in Monocyte/Macrophage Migration

#### 3.5.1. NF-*κ*B

Nuclear factor-kappa B (NF-*κ*B), as an inflammatory hub, is a crucial transcription factor of numerous genes involved in the progression of atherosclerosis, e.g., IL-1*α*, IL-6, IL-8, GM-CSF [63], TNF-*κ* [64], IL-1*β* [65], platelet-activating factor [66], bacterial superantigen [67], taxol [68], IFN*γ* and lipopolysaccharide [69], as well as MCP-1/CCL-2 [70], and macrophage inflammatory protein 1-*α* (MIP1*α*)/CCL-3 [71]. In addition, NF-*κ*B could also increase the secretion of TNF-*α*, which could activate the downstream signaling pathway in turn [72], regulating the process of monocyte/macrophage migration in atherosclerosis.

It has been well established that a number of biochemical factors that could affect the migration of monocytes/macrophages were mediated through the NF-*κ*B signaling pathway. Ye et al. [73] reported CCL18/phosphatidylinositol transfer protein membrane-associated 3 (PITPNM3) could induce the expression of VCAM-1 through NF-*κ*B activation, which could further promote the adhesion and migration of monocytes. Ma et al. [74] demonstrated that palmitic acid (PA) was able to upregulate the expression of MCP-1 through the NF-*κ*B pathway and then induce the migration of monocyte. T. pallidum [75] could enhance the migration of monocyte via the NF-*κ*B signaling pathway by modulating the balance of metalloproteinase (MMP)/tissue inhibitor of metalloproteinases (TIMP). In addition, other factors including Rho-kinase 2 (ROCK2) [76], H_2_O_2_ [77], uric acid (UA) [78], and hypoxia-inducible factor-1 (HIF-1) [79] induced monocytes/macrophages migration via an analogous way. Inhibitors for monocytes migration like lobeglitazone [80], IMM-H007 (H007) [81], lysophosphatidic acid (LPA) [76], *β*-elemene [82], and macrophage migration inhibitory factor (MIF) [83] were all associated with the inactivation of NF-*κ*B signaling pathway.

Moreover, the NF-*κ*B signaling pathway is also involved in the differentiation of monocytes/macrophages in the progression of atherosclerosis. As reported by Binesh et al. [84], notch intracellular domain (NICD) could be regulated by NF-*κ*B inhibition, which in turn down macrophage differentiation afterward. He further proved [85] that diosgenin regulates the differentiation of monocyte not by inhibiting its differentiation to macrophage but inducing its differentiation to M2 macrophage, thus, preventing the promotion of atherosclerosis. In addition, HIF [79] is reported as a crucial factor which could mediate the formation of foam cells by regulating efflux pathways in macrophages through the NF-*κ*B signaling pathway. On the contrary, paeoniflorin (PF) [86] could not only inhibit the expression of MCP-1, affecting the differentiation and migration of monocyte, but also block the foam cell formation via the NF-*κ*B signaling pathway.

#### 3.5.2. oxLDL

Earlier studies demonstrated that oxidized LDL (oxLDL) could promote the differentiation of monocyte [84, 85], the chemotaxis of macrophages [87], but inhibit their egress [88, 89]. Recent studies showed that oxLDL mainly induces monocytes differentiated to M1 macrophage, which are proinflammatory [90] and assumed to have lower migration ability [91]. Studies from Binesh et al. [90] suggested that the differentiation of monocyte and polarization of M1 macrophage are probably associated with the overexpression of NF-*κ*B and NICD. Anand Babu et al. [87] points out that oxLDL provided the oxidative stress in microenvironment stimulates the epithelial cells to overexpress the inflammatory cytokines and NF-*κ*B, which could further improve the infiltration of monocyte/macrophage as mentioned above [92]. Lin et al. [93] reported that oxLDL could promote the secretion of high mobility group proteins B1 (HMGB1) by endothelial cells through caveolin-1 and NF-*κ*B signaling pathway, which can further bind to the HMGB1 receptors and Toll-Like Receptor 4 (TLR4) located on macrophage cell surface, being involved in macrophage recruitment, infiltration, and M1 type polarization. According to Wang's study, oxLDL could affect the migration of macrophages through lectin-like ox-LDL receptor-1 (LOX-1), and this process is associated with the downregulation of calpain-1 and upregulation of calpain-2, a family of calcium-dependent proteases involved in cell migration. In LOX-1 knockout mice, adverse phenomena could be observed, and the accumulation of macrophages in the plaque is reduced significantly. However, the underlying mechanisms are still unclear.

## 4. Biomechanics Involved in Monocyte Migration

It is important to note that the adhesion and migration of monocytes, the earlier step of atherosclerosis initiation, is governed by the expression of adhesion molecules and their ligands as we discussed above, as well as the mechanical interactions between monocytes and endothelial cells. So, factors affecting the biomechanical properties of monocytes and the biomechanical environment that will affect the final monocyte migration behavior are discussed in the following section.

### 4.1. Extrinsic Factors Affecting Monocytes Biomechanical Properties Associated with Migration

#### 4.1.1. Cholesterol

Recently, some studies suggested that the regulation of monocyte migration by some cytokines might be attributed to the change in their biomechanical properties. Saha [94] and his colleagues modified the cellular cholesterol of monocytes by using Methyl-*β*-cyclodextrin (M*β*CD) and M*β*CD-cholesterol complex and investigated changes in spreading behavior, chemotaxis, migration ability, and deformability of monocytes. They found that the migration ability of monocytes was positively correlated with the cholesterol levels, which may be attributed to cholesterol-induced alteration of monocyte biomechanical properties including decreased cellular deformability and chemotaxis while increased cell spreading behavior mediated by cholesterol depletion. Later, they [95] used the same way to examine the relationship between cholesterol and monocyte membrane fluidity as well as the underlying mechanisms. They demonstrated that the cholesterol enrichment resulted in a 1.7-fold decrease in both membrane fluidity and Young's modulus as compared to the untreated control. These phenomena may be associated with lipid rafts disruption and its activation of protein kinase C (PKC) [96, 97], which in turn induces F-actin polymerization, thus, change the cell stiffness [98] and cell spreading ability [99, 100], resulting in changes of the cell deformability. However, they demonstrated in an extended study that changes in biomechanical properties of monocytes were actually due to alterations in the properties of the cell membrane but not the reorganization of cytoskeleton [101]. In all, these studies suggest that increased membrane fluidity due to cholesterol depletion could reduce the nonspecific adhesion force, facilitating monocyte adhesion and migration.

#### 4.1.2. Amyloid-*β* Peptide (A*β*)

In addition to the effects brought by cholesterol, amyloid-*β* peptide (A*β*) could also alter the biomechanical properties of endothelial cells thus enhance the migration of monocytes [102–104]. Askarova et al. [105] observed F-actin polymerization in endothelial cells after A*β* oligomers treatment, which could be reduced after latrunculin A and lovastatin treatment. Moreover, results from atomic force microscopy (AFM) and quantitative immunofluorescence microscopy (QIM) showed that A*β* treatment would increase endothelial cells' adhesion ability and Young's modulus, while decrease the force of membrane tether formation (*F*_mtf_) and stiffness. Interestingly, these changes could also be disrupted by latrunculin A and lovastatin treatments. Therefore, it is reasonable to speculate that A*β* could induce stress fiber formation in endothelial cells, which in turn change the biomechanical behavior of these cells, as well as their interaction with monocytes, affecting monocyte migration across the endothelium layer consequently.

#### 4.1.3. Lipopolysaccharides (LPS)

Ravetto et al. [106] cultured human promyelocytic leukemia HL60 cells and compared the mechanical properties of nontreated cells with lipopolysaccharides (LPS) treated ones. They found that LPS could induce increasement of the elastic compressive modulus of these monocytes by 73-340%, while decrease the cell's shear modulus by 25-88%. Moreover, F-actin polymerization and a structural reorganization were also observed. Taken together, these results suggest that LPS could change monocytes' biomechanical properties by mediating the cytoskeletal reorganization and may cause the promotion of cell adhesion and diapedesis in LPS-induced inflammation.

In all, we could conclude that biochemical factors like cholesterol, A*β*, and LPS could alter the biomechanical properties of monocytes and endothelial cells, including cell deformability, membrane fluidity, cell-cell junctions, and the force of membrane tether formation, by affecting F-actin polymerization and cytoskeletal reorganization. These biomechanical property alterations finally affect the adhesion and migration of monocytes through the endothelial monolayer.

### 4.2. Biomechanical Environment Involved in Monocyte/Macrophage Adhesion, Migration, and Retention

#### 4.2.1. Extracellular Matrix (ECM) Stiffness

Apart from the biomechanical properties per se, the mechanical environment could also affect monocyte adhesion and migration. Studies from Adlerz et al. [107] showed that substrate elasticity could modulate the behavior of monocyte and macrophage in vitro. When culturing on substrates mimicking healthy arterial stiffness (1-5 kPa), the areas of macrophages increase slightly from a sphere, while increase about eight-fold when culturing on stiffer substrates (280 kPa-70 GPa) with enhanced proliferation and migration rates. Moreover, monocytes/macrophages cultured on stiffer substrate will express more adhesion complexes which contribute to the adhesion process [108]. As the stiffness of an atherosclerotic plaque can be ranged from 1 to 250 kPa: lipid (1 kPa), cellular fibrotic (10 kPa), hypocellular fibrotic (60 kPa), elastic (80 kPa) to calcified (250 kPa) areas, we speculate that atherosclerosis progression makes vessel stiffer which in turn facilitates monocytes proliferation and migration. Kothapalli et al. [109] used *β*-aminopropionitrile (BAPN) to resist arterial stiffening in apoE null mice and found the number of macrophages in the atherosclerotic plaque reduced significantly.

#### 4.2.2. Mechanical Stretch of the Vein

The mechanical stretch from the adjacent vein could also regulate the activation of monocytes. Liu's team [110] explanted a nonengineered jugular vein into the abdominal aorta to increase vein tensile stress while using an engineered vein graft with a comparable lower tensile stress as the control. The results showed that the number of activated monocytes was increased in nonengineered veins as compared to the engineered ones; meanwhile, ICAM-1 clustering was also observed.

#### 4.2.3. Blood Flow Induced Shear Stress

Atherosclerosis preferred to occur at the branched or curved arteries with oscillatory (OSS) or low wall shear stress (LSS) [111–113]. This atheroprone shear stress that contributes to the regulation of monocyte adhesion and migration could be described as follows. On one hand, these atheroprone shear stress could upregulate the expression and activation of adhesion molecules (VCAM-1, ICAM-1, and P-selection) and monocyte chemoattractant protein (MCP-1) in endothelial cells [114–116], which will facilitate monocyte recruitment and adhesion [117–119]. On the other hand, LSS or OSS could increase the secretion of proinflammatory mediators such as C-reactive protein, IL-6, GRO-a (or CXCL1), and IP-10 (or CXCL10) in atherosclerotic plaques [120, 121], which could induce M1 macrophage polarization through increasing the expression of RelA (p65 subunit of NF-*κ*B) and c-Jun N-terminal kinase [122] and activate inflammatory mediators in atherosclerosis [123]. In addition, another study showed that both reversing shear stress and LSS could regulate gene expression and promote endothelial proliferation but only reversing shear stress could induce monocyte adhesion [124].

#### 4.2.4. Hypertension

Hypertension is a well-known risk factor for atherosclerosis, and some studies pointed out that blood pressure may also be involved in monocyte/macrophage behavior regulation. As compressive stress, it could reduce the expression of class A scavenger receptors (SRAs), an important lipoprotein receptor in atherosclerosis [125–127], through inducing monocytes SRA transcription and translation in an amplitude-dependent manner [128]. More importantly, the pressure-induced cyclic strain could increase the expression of ICAM-1 and enhance the attachment between monocytes and endothelial cells [129, 130].

It should be noted that monocytes and macrophages are highly sensitive to hypertension-related biomechanical stimulation. Only small stimuli could upregulate the expression of immediate-early genes, such as c-fos and c-jun, but not ets-1 [131] and IEX-1 gene in monocytes [132]. What is more, the deformation of monocytes and macrophages could upregulate the expression of PU.1 [131], and its interactions with c-jun play an important role in the regulation of macrophage differentiation [133–136]. In addition, hypertension-related mechanical stress could regulate the secretion of matrix metalloproteinase (MMP) in monocytes, such as MMP-1, MMP-3, and TIMP-1, but not MMP-9 [131], as well as MCP-1 and IL-8 in endothelial cells [132, 137–139].

It has been shown that statins for atherosclerosis therapy could also lower blood pressure [140–142]. Hence, we can speculate that the effectiveness of statins for atherosclerosis therapy may partially be attributed to its pressure-lowering effect which in turn induced a positive mechanical environment to regulate the behavior of monocyte/macrophages, sustaining the stability of the plaque.

## 5. Potential Therapeutic Approaches Targeting Plaque Macrophages

Therapeutic strategies that reduce macrophage recruitment to atherosclerotic plaques or promote macrophage apoptosis, efferocytosis, or emigration, have been proposed to be efficient on atherosclerosis in both animal models and a few clinical experiments. It may be desirable to selectively deliver small interfering RNAs (siRNAs) or other small molecules to plaques by using nanoparticles or reconstituted lipoproteins.

### 5.1. Therapeutic Approaches Targeting CCL2 and CCR2

Therapeutic approaches for blocking CCR2/CCL2-mediated monocytes recruitment include using siRNAs [143], monoclonal antibodies, CCR2 antagonists, pharmacological inhibition [144], and MCP-1 inhibitors.

#### 5.1.1. siRNAs

Small-interfering RNAs (siRNAs) are proved to be beneficial in myocardial infarction healing in atherosclerosis-prone animal models by Leuschner et al. [145, 146]. They reported that administration of nanoparticle-encapsulated siRNA targeting CCR2 by silencing reduces inflammation in autoimmune myocarditis in vivo, which was associated with the reduction in Lys6C^high^ monocytes at the inflammation site and the inhibition of migration of bone marrow granulocyte macrophage progenitors into the blood.

#### 5.1.2. Monoclonal Antibodies

Several monoclonal antibodies for CCR2 are under development to treat cardiovascular disease caused by atherosclerosis, one famous star of which is MLN-1202. One controlled clinical study was performed to test the therapeutic effect of MLN-1202 on cardiovascular diseases, in which the circulating levels of high-sensitivity C-reactive protein, as the established biomarker of inflammation associated with coronary artery disease, were evaluated [147]. Results demonstrated that CCR2 blockage by MLN-1202 indeed reduced serum C-reactive protein. Moreover, levels of serum CCL2 were enhanced, while levels of CCL2 decreased in circulating monocytes. These phenomena may be attributed to the interacts of MLN-1202 and CCR2, which inhibits CCL2 binding, thus, receptor-mediated clearance of CCL2, as the major way of CCL2 level regulation in humans, was blocked. In mice, CCR2 antagonists have also been shown to prevent egress of CCR2-sensitive monocytes from the bone marrow. However, the underlying mechanism is not clear.

#### 5.1.3. Antagonists: Propagermanium (PG), TLK-19705, GSK1344386B, and PA508

Drug propagermanium (PG), a 3-oxygermylpropionic acid polymer, is known in Japan as a medicine for chronic hepatitis B, while it is also a CCR2 antagonist. It has been demonstrated that PG can reduce atherosclerosis in apolipoprotein E knockout mice via inhibition of macrophage infiltration [148]. It should be mentioned that, unlike antibodies, PG has a unique CCR2 antagonistic activity, since it selectively inhibits CCR2-mediated monocyte migration without affecting CCL2/CCR2 binding or CCL2-stimulated Ca^2+^ mobilization [149].

Based on this drug, Okamoto et al. [149] developed a smaller but similar molecule, named TLK-19705, which is also a promising drug for the treatment of atherosclerosis. Results from animal models showed that the area of atherosclerotic lesion indeed remarkably decreased. In addition, GSK1344386B as another CCR2 antagonist also showed the ability to reduce the macrophage content in the atherosclerotic plaque by selective CCR2 inhibition [150]. Moreover, CCL2 mutant PA508, as a CCL2 competitor, can combine with CCR2 without functional activation and, thus, reduce CCL2-mediated inflammatory monocyte recruitment [151].

#### 5.1.4. CCL2 Antibodies

Neutralizing antibodies were used to block CCL2 in animal models [152], and they did inhibit macrophage infiltration but they brought some potential side effects such as neovascularization disorders and impaired immune system function.

In all, blocking monocyte recruitment via CCR2 seems to be an effective strategy in atherosclerosis progression. However, attention should be paid carefully since CCR2 is widely expressed in lymphocytes, which means therapeutic strategies for monocyte recruitment inhibition may at the same time bring side effects on the immune system. In addition, side effects, off-target effects, and additional impacts on other receptors should also be considered when it comes to treatment methods [153]. Indeed, some treatments can be toxic [145], for example, the TAK-779, a dual antagonist of CCR2 and CCR5, showed inhibitory effects not only on CCR2 but also on CCL5/CCR5. Moreover, timing may also be an issue for targeting chemokine receptors. CCL2 plays an important role in the initial stage of atherosclerosis, and the blockage of CCL2/CCR2 at the beginning can inhibit monocyte recruitment, while it inevitably affects the transformation of monocytes into M2 stable macrophages in the later stage, thus, may further affect the regression of atherosclerosis [14].

The targeting therapies described above are summarized in Table 2.

### 5.2. Therapeutic Approaches Targeting CCR7 and Semaphorin3A

Recent studies in mice showed that low-density lipoprotein receptor-related protein 1 depletion in macrophages accelerated atherosclerotic plaque formation, due to enhanced apoptosis of the cells, decreased efferocytosis, and exaggerated transition of them to the inflammatory M1 phenotype. However, upregulation of CCR7 will unexpectedly accelerate atherosclerosis regression in a bone marrow transplantation experiment [154]. Several factors affecting the expression of CCR7 have been proposed [156]. It has been proved that in ox-LDL-treated Raw264.7 macrophages, CCR7 gene expression was downregulated [45] at both mRNA and protein levels. Moreover, it has been observed in the transplantation mice model that the plasma non-HDL levels were decreased, while HDL levels restored [157], which was accompanied by a reduced content of plaque CD68(+) cells (mainly macrophages) resulting from the enhanced emigration of macrophages and induction of their chemokine receptor CCR7. Notably, statins were also proposed to induce CCR7 expression in vivo and promote plaque regression via emigration of CD68+ cells in a CCR7-dependent manner [155]. In addition, UNC5b as the receptor of Netrin-1 has been also shown to be associated with CCR7 expression that decreased after pcDNA3.1-UNC5b plasmid treatment, while increased after treated with siUNC5b [45].

Studies showed that local delivery of Semaphorin 3A may act as a novel therapeutic option to prevent in-stent restenosis as it suppressed neointimal hyperplasia after vascular injury in vivo [158]. This could be explained by its ability to inhibit smooth muscle cell migration. However, its potential role in atherosclerosis progression is waiting to be discovered.

## 6. Conclusion and Future Directions

According to the “response to injury” hypothesis, the recruitment and migration of monocytes play important roles in atherosclerosis progression. Several cytokines/chemokines have been reported to be involved in the regulation of monocyte recruitment like CCL2/CCR2, CCL5/CCR5, and CX3CL1/CX3CR1, the egress of these cells from the plaque like CCR7/CCL19/CCL21, and migration blocking like Netrin-1 and Semaphorin3A. Till now, some of these cytokines/chemokines have been well studied, like CCR2/CCL2 and CCR7, but some have just begun to be known, such as Netrin-1 and Semaphorin3A. More importantly, during the processes of monocyte recruitment and migration, biomechanics are also involved. On one hand, the biomechanical properties of the monocytes per se like the membrane fluidity, the deformability, and stiffness will be affected by some extrinsic or intrinsic factors like cholesterol, amyloid-*β* peptide (A*β*), and lipopolysaccharides (LPS). On the other hand, monocytes situated biomechanical environment such as the stiffness of the extracellular matrix, stretch from the neighboring vein, blood flow-induced shear stress, and hypertension, are all involved in monocytes regulation. Unfortunately, when we come to seek therapeutic approaches to encourage macrophages to egress from the established plaque, we just put our eyes on the reported cytokines. Seeking more possible factors that will affect the biomechanics of monocytes and finding its underlying mechanism will be helpful in future studies.

## Figures and Tables

**Table 1:** Cytokines involved in the progression of atherosclerosis.

Involved stage	Component	Characteristics	Mechanisms in atherosclerosis	References
Recruitment	CCL2	Small proteins with four completely conserved cysteine residues, expressed by a variety of cell types	Guide Ly6C^high^ monocytes to migrate into the subendothelial space and promote the bone marrow release of Ly6C^high^ monocytes to the blood circulation	[15–17, 25–27]
	CCL5	Proteins secreted by monocyte, macrophages, T cells, and smooth muscle cells	Play a critical role in Ly6C^low^ monocytes' adhesion and recruitment	[13, 30, 33, 34]
	CXC3L1	Expressed by endothelial cells as a membrane-bound protein	Allow firm adhesion of monocytes on endothelial and smooth muscle cells independently of integrin activation and improve their survival from the bone marrow	[13, 14, 30–32]

Egress from the plaque	CCL19	Produced by stromal cells within primary and secondary lymphoid organs	Promote chemotactic migration of the macrophage to egress from the plaque	[38–41]
	CCL21	Produced by stromal cells within primary and secondary lymphoid organs and lymphatic endothelial cells (LECs) in peripheral tissues	Promote chemotactic migration of macrophage to egress from the plaque	[38–43]

Migration inhibition	Netrin-1	Neuronal guidance molecules expressed in many cells such as endothelial cells and foam cells	Inhibit the migration of monocytes into the intima and egress of macrophage from the plaque	[5, 9, 14, 47, 49, 51, 52]
	Semaphorin-3A	Secreted proteins expressed by endothelial cells and immune cells like macrophages	Act as a barrier to prevent monocyte adhesion and migration into the arterial intima during the early stage of atherogenesis	[57–60]
	Semaphorin-3E	Secreted, highly conserved, and matrix-associated proteins by macrophages, especially M1 macrophages	Inhibit macrophages migration and egress of macrophage from the plaque	[61, 62]

**Table 2:** Potential therapeutic approaches targeting plaque macrophages.

Target	Method/drugs		Possible mechanisms	References
CCR2	Nanoparticle-encapsulated siRNA		Reducing Lys6C^high^ monocytes at the inflammation site and the inhibition of migration of bone marrow granulocyte macrophage progenitors into the blood	[145, 146]
	Monoclonal antibodies: MLN-1202		Reducing serum C-reactive protein and preventing egress of CCR2-sensitive monocytes from the bone marrow	[147]
	Antagonist	Propagermanium	Selectively inhibiting CCR2-mediated monocytes migration without affecting CCL2/CCR2 binding or CCL2-stimulated Ca^2+^ mobilization	[148, 149]
		TLK-19705	A smaller but similar molecule like propagermanium	[149]
		GSK1344386B	Selective CCR2 inhibition	[150]
		PA508	CCL2 mutant as a CCL2 competitor, combine with CCR2 without functionally activation	[151]

CCL2	Antibodies		Inhibiting macrophage infiltration while bringing some potential side effects such as neovascularization disorders and impaired immune system function	[152]

CCR7	Statins or bone marrow transplantation		Upregulating CCR7, promoting the egress of macrophages from the plaque, and helping it return to the lymphoid tissue	[154, 155]

## Data Availability

No data were used to support this study.

## Additional Points

*Key Messages*. The entry and egress of monocytes into and from the plaques determine the progression of atherosclerosis.

Multiple cytokines and receptors are involved in monocyte recruitment such as CCL2/CCR2, CCL5/CCR5, and CX3CL1/CX3CR1, and the egress of macrophages from the plaque like CCR7/CCL19/CCL21. Some neural guidance molecules such as Netrin-1 and Semaphorin 3E also showed an inhibitory effect on monocyte migration.

Factors affecting the biomechanical properties of monocytes, like cholesterol, amyloid-*β* peptide (A*β*), and lipopolysaccharides (LPS), and the biomechanical environment that will affect the monocyte migration behavior.
